# *W3* Is a New Wax Locus That Is Essential for Biosynthesis of β-Diketone, Development of Glaucousness, and Reduction of Cuticle Permeability in Common Wheat

**DOI:** 10.1371/journal.pone.0140524

**Published:** 2015-10-15

**Authors:** Zhengzhi Zhang, Wenjie Wei, Huilan Zhu, Ghana S. Challa, Caili Bi, Harold N. Trick, Wanlong Li

**Affiliations:** 1 Department of Biology and Microbiology, South Dakota State University, Brookings, South Dakota, 57007, United States of America; 2 Department of Plant Pathology, Kansas State University, Manhattan, Kansas, 66506, United States of America; Institute of Genetics and Developmental Biology, Chinese Academy of Sciences, CHINA

## Abstract

The cuticle plays important roles in plant development, growth and defense against biotic and abiotic attacks. Crystallized epicuticular wax, the outermost layer of cuticle, is visible as white-bluish glaucousness. In crops like barley and wheat, glaucousness is trait of adaption to the dry and hot cultivation conditions, and hentriacontane-14,16-dione (β-diketone) and its hydroxy derivatives are the major and unique components of cuticular wax in the upper parts of adult plants. But their biosynthetic pathway and physiological role largely remain unknown. In the present research, we identified a novel wax mutant in wheat cultivar Bobwhite. The mutation is not allelic to the known wax production gene loci *W1* and *W2*, and designated as *W*3 accordingly. Genetic analysis localized *W3* on chromosome arm 2BS. The *w3* mutation reduced 99% of β-diketones, which account for 63.3% of the total wax load of the wild-type. *W3* is necessary for β-diketone synthesis, but has a different effect on β-diketone hydroxylation because the hydroxy-β-diketones to β-diketone ratio increased 11-fold in the *w3* mutant. Loss of β-diketones caused failure to form glaucousness and significant increase of cuticle permeability in terms of water loss and chlorophyll efflux in the *w3* mutant. Transcription of 23 cuticle genes from five functional groups was altered in the *w3* mutant, 19 down-regulated and four up-regulated, suggesting a possibility that *W3* encodes a transcription regulator coordinating expression of cuticle genes. Biosynthesis of β-diketones in wheat and their implications in glaucousness formation and drought and heat tolerance were discussed.

**Key Message:**

*W3* is essential for β-diketone biosynthesis but suppresses its hydroxylation. Loss-of-function mutation *w3* significantly increased cuticle permeability in terms of water loss and chlorophyll efflux.

## Introduction

During transition from water to land colonization, plants developed an array of mechanisms for adaptation to the desiccation environment. One of these mechanisms is deposition of cuticle, a hydrophobic coat, to cover the aerial organ surfaces. In addition to function in protecting water loss, cuticle, as the interface between the sessile plants and their environment, plays important roles in plant defense against heat, UV radiation, pathogen and insect attacks [1]. Cuticle consists of the fundamental framework of cutin and intracuticular wax inserted in it and epicuticular wax overlaid on them. Cutin is the cell wall-bounded ester polymer of hydroxy fatty acids [2–4], and waxes are the very long chain fatty acids (VLCFAs) and their derivatives including alcohols, aldehydes, alkanes, ketones and wax esters [5]. Variation in epicuticular wax composition causes changes in plant appearance: glaucous or nonglaucous. Glaucousness, the bluish-white look, is the visible form of densely arrayed wax crystals.

In wax biosynthesis, *de novo* C_16_ and C_18_ fatty acids, the precursors, are first elongated into VLCFAs (C_20_ to C_34_) by fatty acyl elongase (FAE) complex. The VLCFAs can be converted to primary alcohols and further esterified with the C_16_ fatty acids into wax esters via the acyl reduction pathway [6–8] and converted to alkanes, aldehydes, secondary alcohols and ketones through decarbonylation pathway [9–11]. All the wax species are synthesized in epidermal cells, transported extracellular and deposited to cuticle. Wax synthesis and export are under tight developmental and environmental regulations. Molecular identification of the genes coding for the wax biosynthetic enzymes, wax transporters and wax regulators in the model plant Arabidopsis provided genetic and molecular supports to the VLCFA and associated pathways (reviewed in [12–14].

The VLCFA and associated pathways are conserved in higher plants, and many of their components of these pathways have also been identified in the grass genomes. Maize wax genes *GLOSSY1* (*GL1*) [15, 16], *GL2* [17], *GL4* [18] and *GL8* [19, 20] are homologous to *ECERIFERUM3* (*CER3*), *CER2*, *CER6* and *β-keto acyl-CoA reductase 1* (*KCR1*) of Arabidopsis, respectively. A T-DNA insertion mutant in a *CER6* homolog reduced leaf wax crystal density in rice [21]. Maize AP2 transcription factor GL15 involves in the transition from juvenile to adult leaf identity including wax composition [22], and HD-ZIP IV family transcription factor OCL1 regulates cuticular wax biosynthesis by activation of a fatty acyl-CoA reductase (FAR) and a putative wax transporter [23, 24].

In addition to the VLCFA and associated pathways, a diverse of plants, including barley and wheat (*Triticum* L.), employ another parallel wax biosynthetic pathway for biosynthesis of hentriacontane-14,16-dione (also known as β-diketone) and its derivatives. In wheat, the VLCFA pathways are active in the vegetative stage, but the β-diketone pathway predominates in the reproductive stage [25]. It is believed that β-diketone is synthesized using C_14_ and C_16_ fatty acids as precursors [26]. Wax profiling of barley mutants suggests that *cer-q*, *cer-c* and *cer-u* mutants respectively define the initial condensing reaction, subsequent chain extensions and hydroxylation [27], but little is known about the genetic components of this pathway. In common wheat (*T*. *aestivum* L., genomes AABBDD), two wax inhibitors and two wax production genes underlie the glaucousness variations: *Iw1* on chromosome arm 2BS [28–31], *Iw2* on 2DS [30–33], *W1* on 2BS [30, 34] and *W2* on 2DS [30]. In durum wheat (*T*. *turgidum* subsp. *durum*, genomes AABB), a third wax inhibitor *Iw3* is located in the distal region of chromosome arm 1BS [35, 36]. We previously characterized a set of wheat near isogenic lines (NILs) differing at *W1*, *W2*, *Iw1* and *Iw2* loci and durum NILs differing at the *Iw3* locus, and found that loss of both functional alleles of the *W* genes or presence of either *Iw* gene causes depletion of β-diketones and nonglaucous phenotype. While elimination of β-diketones is compensated by increase of aldehydes and primary alcohols in the *Iw1* and *Iw2* NILs [37], *Iw3* inhibits β-diketone, reduces primary alcohols but increase aldehyde and alkanes in the glume wax [36]. Although expression of a *FAR1* homolog was maintained at high level in the *Iw* NILs, no match was found between expression of 72 wax genes and β-diketone distribution pattern [36, 37]. All this suggests that β-diketones are synthesized through a not-yet-identified pathway.

We recently identified a nonglaucous (NG) mutant in Bobwhite (BW) wheat. Compared to the wild type BW, the mutant lost 63% of total wax load and almost all of the β-diketones, and showed a significant increase of water loss. Genetic and molecular analyses indicated that the mutant gene is not allelic to either *W1* or *W2* and expression of numerous cuticle genes of five different pathways was significantly down regulated. Here we report the results and their implications in Triticeae cuticle biosynthesis and drought tolerance.

## Results

### Genetic Analysis of the Wax Mutant

**Fig 1:**
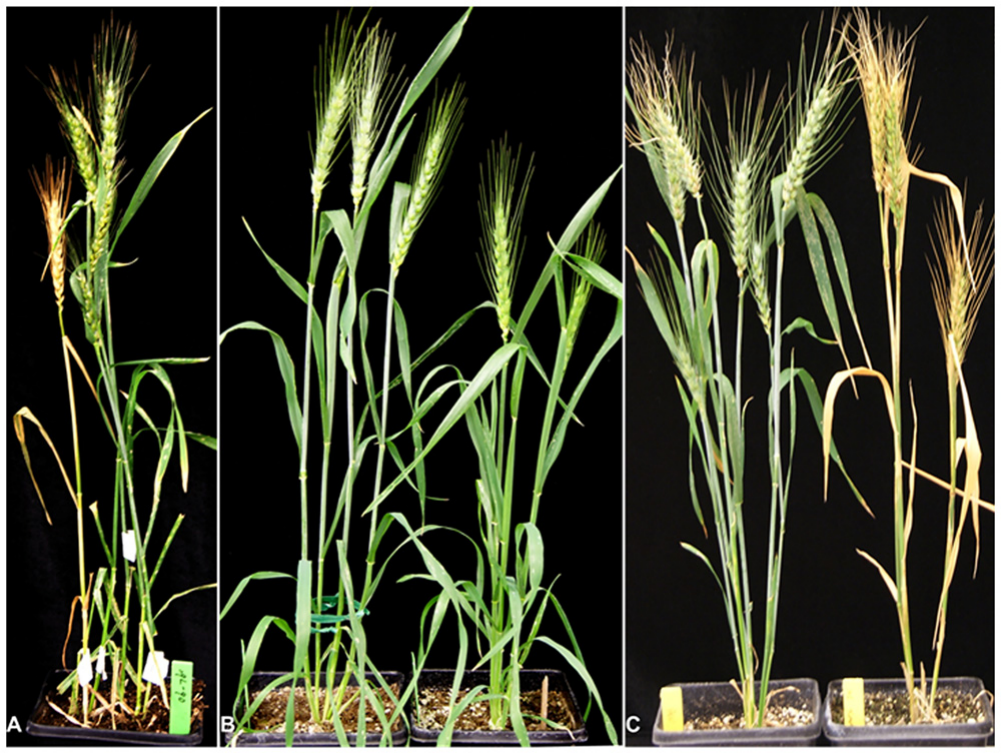
Phenotype of the nonglaucous mutant. (***A***) The mutant was found in a small F_2_ population derived from a cross between two RNAi transgenic plants in BW background due to premature drying out. (***B***) Adult plants of BW (left) and the nonglaucous mutant (right) at anthesis. (***C***) Adult plants of BW (left) and the nonglaucous mutant (right) at grain-filling stage.

During intercrossing BW transgenic plants to combine the different RNAi transgenes, a NG plant was found in a small T_1_ population of the transgenic line #056 in the spring of 2010. It dried out prematurely (Fig 1A). We recovered two NG plants, NG1 and NG2, from the T_2_ populations derived from the glaucous T_1_ plants of the same pot. These NG plants had much lower level of glaucousness in leaves, sheaths, peduncle, and spikes as compared to the wild type BW (Fig 1B). The mutant plants died prematurely again in the late spring of 2011 although they were well watered (Fig 1C) probably due to the high temperature.

**Fig 2:**
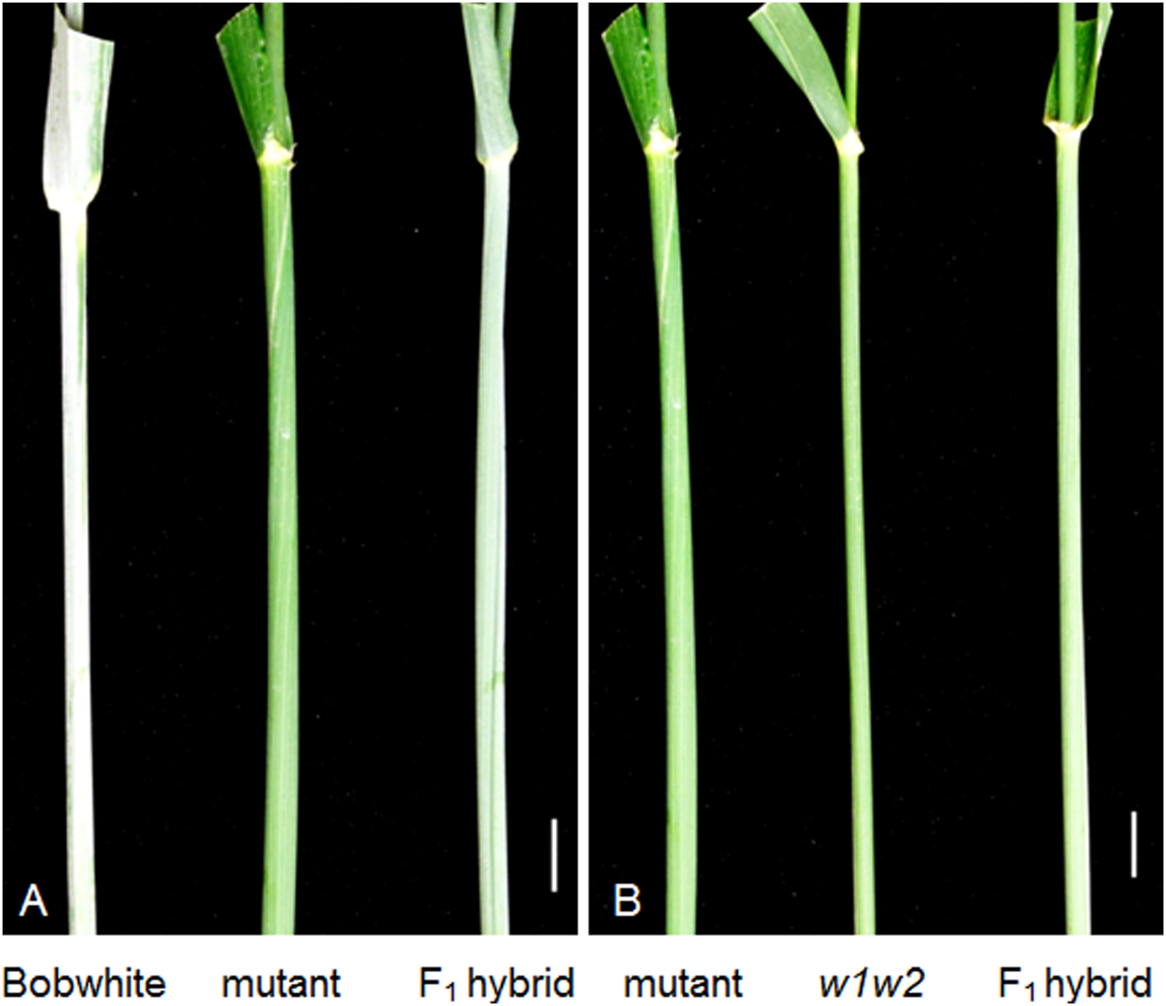
Genetic analysis of *w3* mutant. (***A***) The F_1_ hybrid between BW and the nonglaucous mutant is intermediate between its parents in glaucousness intensity. (***B***) The F_1_ hybrid between the mutant and *w1w2* double recessive line was glaucous. The scale bars indicate 1 cm.

We started genetic analysis of this presumable NG mutant by crossing it with the wild type BW. The F_1_ plants were glaucous but their glaucousness level was intermediate between BW and the NG mutant (Fig 2A). A population of 361 F_2_ individuals segregated into 265 glaucous and 96 nonglaucous, which fits the 3 (glaucous) to 1 (nonglaucous) ratio (*P* = 0.38812; Table 1), indicating that a single-gene mutation underlies the nonglaucous phenotype. To test if the mutation was due to transgene insertion, we screened 38 nonglaucous F_2_ individuals derived from the cross between NG1 and BW for the transformation selection marker *bar* gene and RNAi transgene constructs [38] by targeting at the *Ubq-bar* and *gus-nos* junctions, respectively. Result showed that eight plants were negative for both the *Ubq-bar* and *gus-nos* junctions, and 7 additional plants were negative for the *gus-nos* junction. Furthermore, NG2 contained neither *Ubq-bar* nor *gus-nos* (S1 Fig). These data indicate that one *bar* locus was not linked with the RNAi locus in NG1 and that the nonglaucous mutation is independent of the transgene insertions.

**Table 1:** Phenotypes and segregations of glaucousness in hybrids and their progenies.

Crosses	# of F_1_	F_1_ Plant	F_2_ segregation	*P* values	*P* values	*P* values	*P* values
	plants	phenotype	(gl : ng)*	(3 : 1)	(15 : 1)	(9 : 7)	(1 : 1)
*w3* x BW	5	glaucous	106:38	0.70032	1.8 x 10^−23^	2.7 x 10^−5^	1.3 x 10^−10^
*w3* x CS-TDIC 2B	5	glaucous	265:96	0.38812	5.9 x 10^−56^	7.9 x 10^−11^	15 x 10^−18^
*w1w2* x *w3*	5	glaucous	141:129	5.4 x 10^−18^	7.6 x 10−^175^	0.18217	0.46477
*w1w2* x BW	5	glaucous	133:11	1.5 x 10^−6^	0.49112	2.4 x 10^−24^	1.2 x 10^−81^
*w3* x CS 2BS-1	6	nonglaucous	0:71	3.0 x 10^−48^	1.3 x 10^−233^	1.2 x 10^−21^	3.6 x 10^−17^
*w3* x CS 2BS-2	6	nonglaucous	0:71	3.0 x 10^−48^	1.3 x 10^−233^	1.2 x 10^−21^	3.6 x 10^−17^
*w3* x CS 2BS-3	6	nonglaucous	0:72	6.7 x 10^−49^	7.3 x 10^−237^	6.5 x 10^−22^	2.6 x 10^−17^
*w3* x CS 2BS-5	6	nonglaucous	0:68	2.8 x 10^−46^	8.1x 10^−224^	8.7 x 10^−21^	1.6 x 10^−16^
*w3* x CS 2BS-10	6	nonglaucous	0:72	6.7 x 10^−49^	7.3 x 10^−237^	6.5 x 10^−22^	2.6 x 10^−17^
*w3* x CS 2BS-14	6	nonglaucous	0:35	2.3 x 10^−34^	3.5 x 10^−116^	2.0 x 10^−11^	3.3 x 10^−9^

*gl, glaucous; ng, nonglaucous.

**Fig 3:**
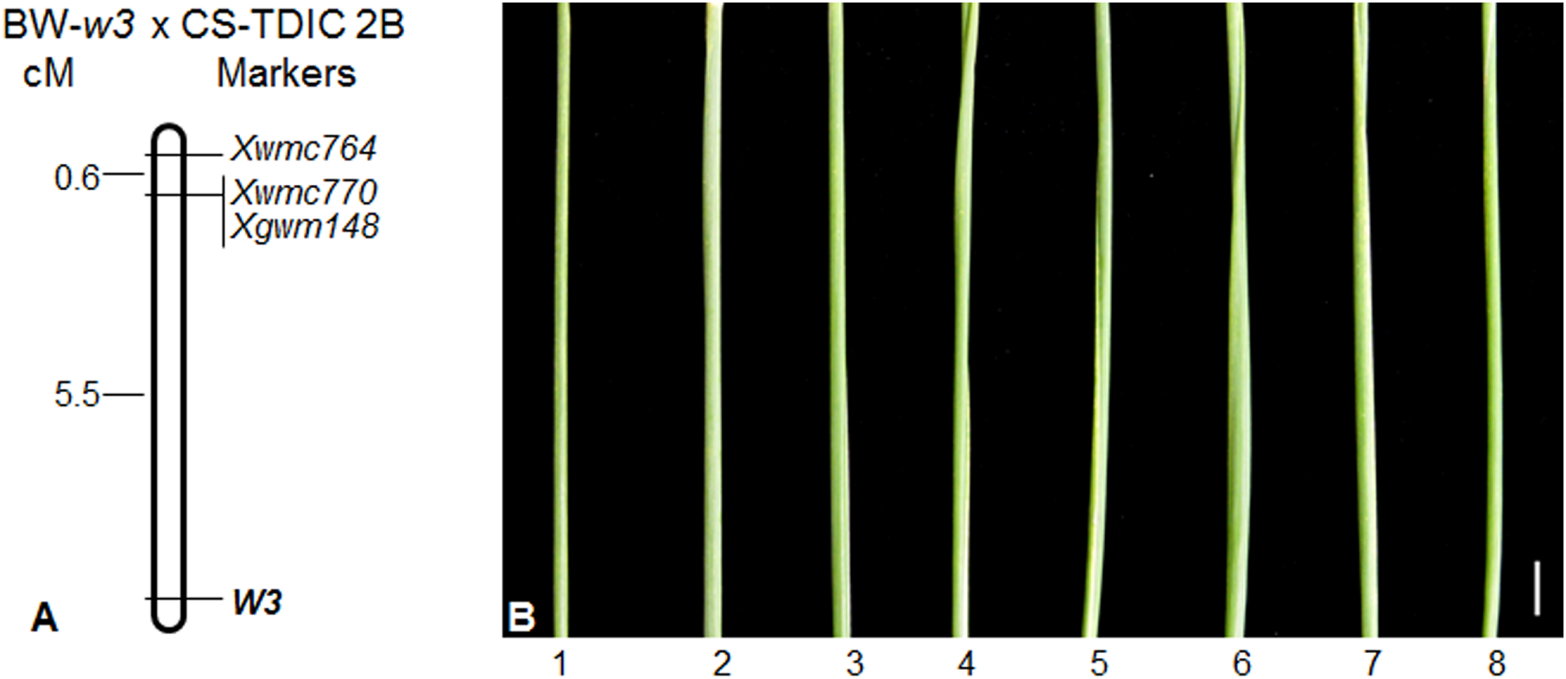
Chromosomal localization of the *W3* locus. (***A***) Molecular mapping of the *W3* locus on the chromosome arm 2BS. The markers are listed at the right side of the map, and genetic distances (cM) between the marker loci are indicated at the left side of the map. The *W3* locus is indicated in bold. The top of the map is towards the telomere and the bottom is towards the centromere. (***B***) The peduncles of CS N2B-T2D (1), F_1_ hybrids of NG2 with N2B-T2D (2), with deletion line 2BS-1 (3), with 2BS-2 (4), with 2BS-3 (5), with 2BS-5 (6), with 2BS-10 (7) and with 2BS-14 (8). All the flag-leaf sheaths are nonglaucous. The scale bar indicate 1 cm.

To test if the nonglaucous mutation is allelic to the known wax production genes in wheat, we crossed S-615 *w1w2* near isogenic line (NIL), which is nonglaucous due to loss-of-function at the functionally redundant *W1* and *W2* loci [37], with BW and the NG2 mutant line. The F_2_ population derived from the cross between S615-*w1w2* and BW segregated into 133 glaucous and 11 nonglaucous plants, well-fitting the 15 to 1 ratio (*P* = 0.49112; Table 1) and indicating that BW carries both *W1* and *W2* functional alleles. The F_1_ hybrid plants between S615-*w1w2* and NG2 carry glaucousness at low intensity (Fig 2B), indicating that the nonglaucous mutation in BW is not allelic to either *W1* or *W2*, but its wild type allele in S615-*w1w2* complements to the functional alleles of the *W1* and *W2* loci carried by BW. According to the recommended rules for gene symbolization in wheat [39], we designated this new wax gene locus as *W3* and the mutant as *w*3. A population of 270 F_2_ plants derived from the cross between the *w3* mutant and the *w1w2* NIL segregated into 141 glaucous and 129 nonglaucous, which fits a 1 to 1 (*P* = 0.46477) or 9 to 7 ratio (*P* = 0.18217; Table 1) and is more similar to the two-locus segregation ratios than a three-locus segregation. These results corroborate that *W3* is not homologous to the known wax gene loci *W1* and *W2* and suggested that *W3* is most probably linked with the *W1* or *W2* locus. To identify the chromosomal location of the *W3* locus, we developed a mapping population from a cross between the *w3* mutant and Chinese Spring (CS)-*T*. *turgidum* subsp. *dicoccoides* 2B substitution line (CS-TDIC 2B) and genotyped the 82 NG F_2_ individuals (168 gametes) using 2BS and 2DS SSR markers for detection of marker-*W3* linkage. The result showed that *W3* is linked with the 2BS SSR markers and 5.5 cM proximal to the marker loci *Xwmc770* and *Xgwm148* (Fig 3A).

We also crossed the *w3* mutant with CS 2BS deletion lines (2BS-1, -2, -3, -5, -10 and -14). All these deletion lines and nullisomic 2B-tetrasomic 2D (N2B-T2D), in which lack of one pair of chromosome 2B is compensated by two pairs of chromosome 2D, are nonglaucous due to missing the *W1* locus. The F_1_ hybrid plants between the *w3* mutant and the 2BS deletion lines were nonglaucous similar to N2B-T2D, or carried nearly undetectable glaucousness (Fig 3B). No glaucous individuals were observed in their F_2_ populations (Table 1). This result corroborates that the *W3* locus is located in the distal region of chromosome arm 2BS.

Furthermore, we genotyped BW and the *w3* mutant using SSR markers located in the distal regions of the 42 wheat chromosome arms on wheat genetic maps. All 42 SSR markers were positive and detected monomorphism (S1 Table). This indicated BW and the *w3* mutant shares the same genetic background.

### Wax Morphology

**Fig 4:**
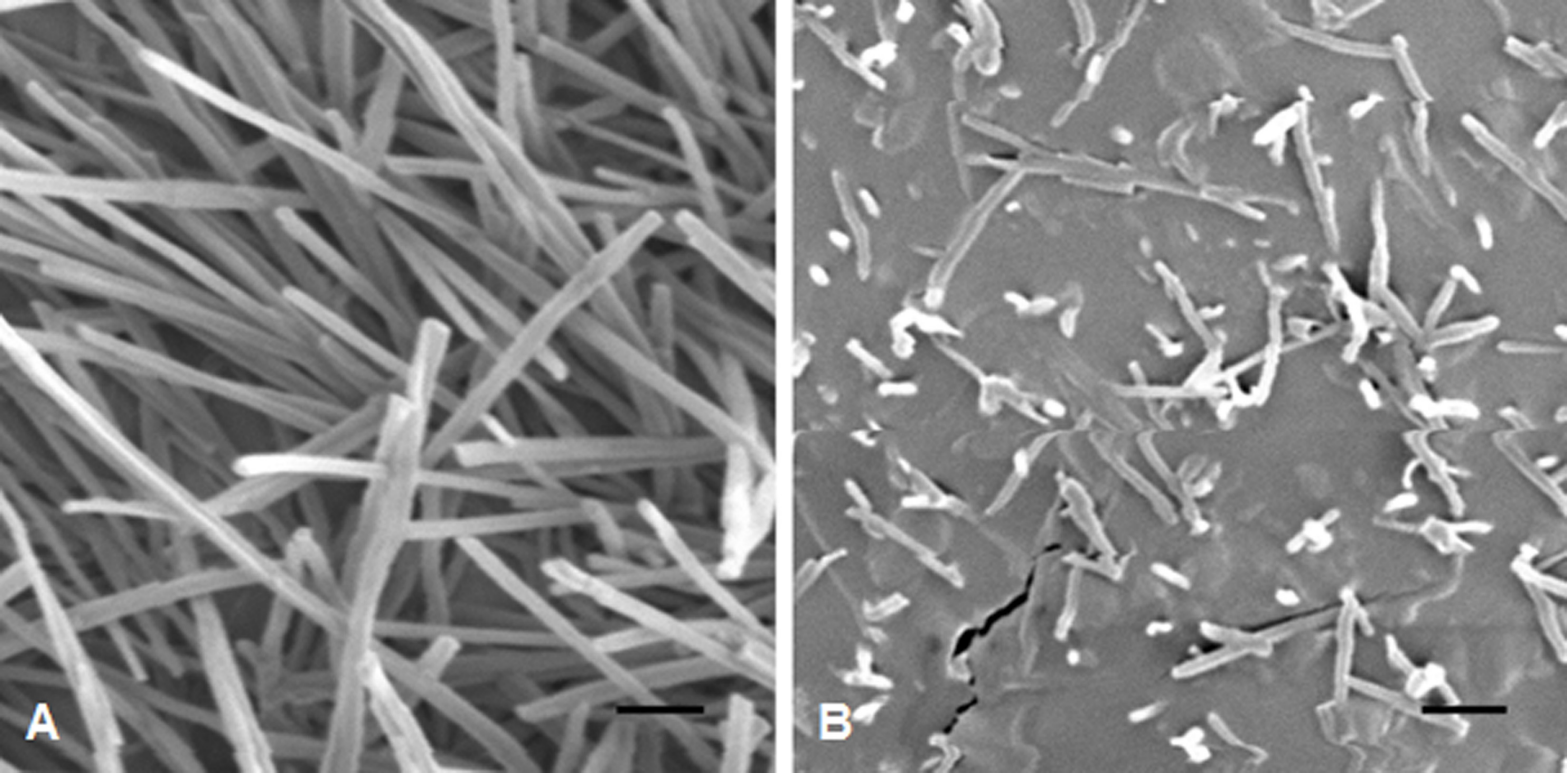
SEM micrographs of cuticle surfaces of flag leaf sheaths. (***A***) BW and (***B***) *w3* mutant. The scale bars indicated 1 μm.

At stage F10.5.1, wheat plants are flowering, and glaucousness is fully developed. We inspected the cuticle surfaces of flag leaf sheaths of BW and *w3* mutant under a scanning electron microscope (SEM) at this developmental stage. The electron micrographs showed clear differences between BW and *w3* mutant (Fig 4). In BW, cuticle surface was covered with thick meshwork of long wax crystal tubes (Fig 4A). In contrast, short wax bodies were appressed to the leaf cuticle surface of *w3* mutant at low density (Fig 4B). This result indicated that the *W3* locus has a major effect on cuticle, epicuticular wax in particular, development. Considering that the great contrast in wax crystal morphology and density between BW and *w3* mutant was found in the flag leaf sheath, we used this tissue for measuring cuticle permeability, profiling composition of the cuticular waxes and quantifying transcription of cuticle genes.

### Cuticle Permeability

**Fig 5:**
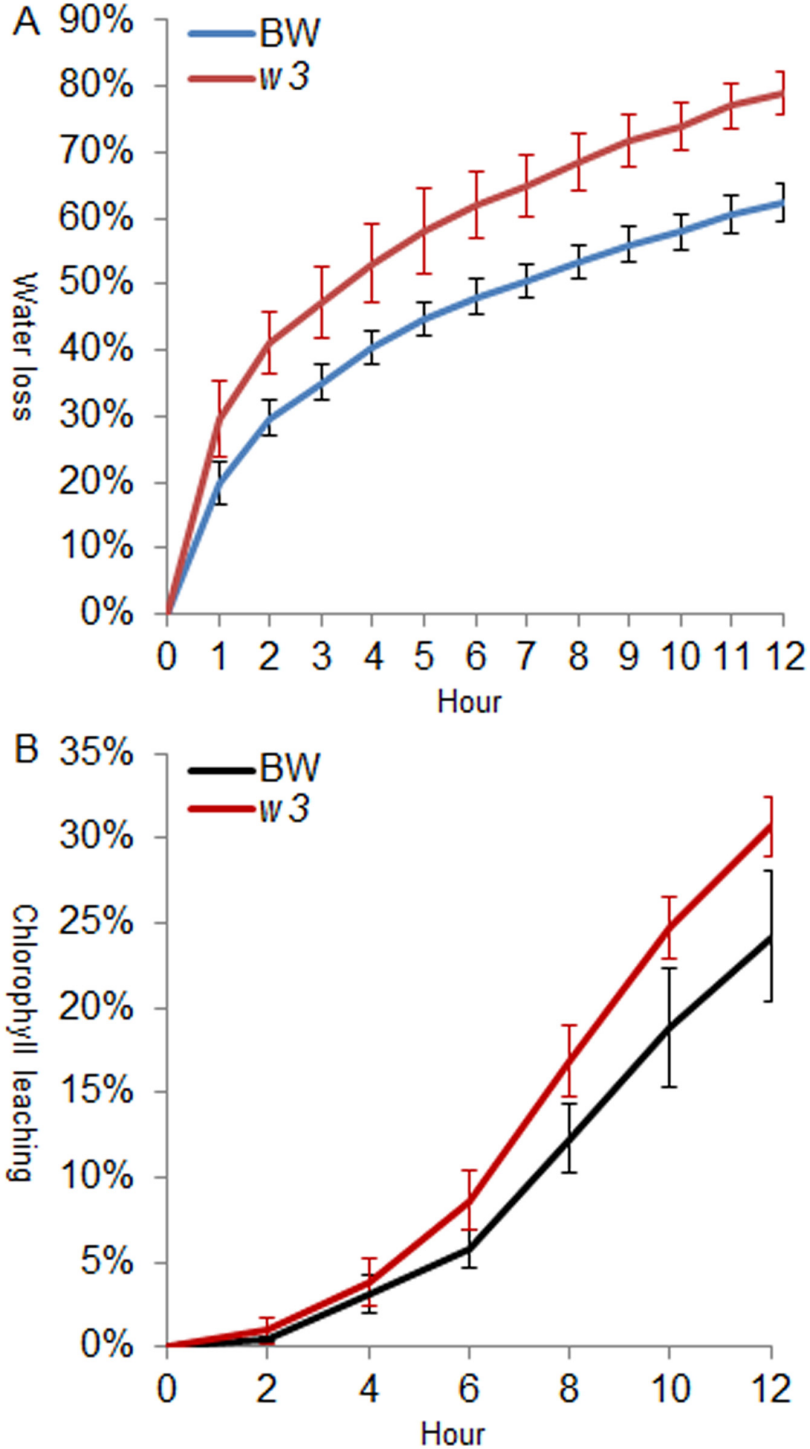
Analysis of cuticle permeability of BW and *w3* mutant. Cuticle permeability was evaluated by air drying at room temperature (***A***) and by chlorophyll leaching in 80% ethanol (***B***). The numbers on the x-axes represent hours of treatment. Water loss or chlorophyll leaching at each time point is represented on the y-axes as percentages of the total water content or total chlorophyll content in the tissue. Measurements taken from six individuals were averaged.

We measured the cuticle permeability in two experiments using the flag-leaf sheath: water loss in air and chlorophyll leaching in 80% ethanol. In the first experiment, significantly higher water loss rate was observed in the *w3* mutant 1 h after detachment (*P* < 0.03303) and incremented throughout the time course (Fig 5A). We inspected stomata, and no significant difference was found in stomata density between BW and *w3* mutant, ~60 stomata in one view field of 10x20 magnification under microscope. The stomata were closed within one hour after detachment. This suggests that the difference in water loss rate is attributed to cuticle permeability. In the second experiment, *w3* mutant showed significantly higher chlorophyll efflux rate from the sixth to eighth hour of treatment (*P* < 0.02491; Fig 5B). Both lines were similar in total chlorophyll content after 48-hour extraction (*P* = 0.80435). This corroborates the conclusion from the water loss experiment that change of wax crystal morphology and density led to increase of cuticle permeability in *w3* mutant.

**Fig 6:**
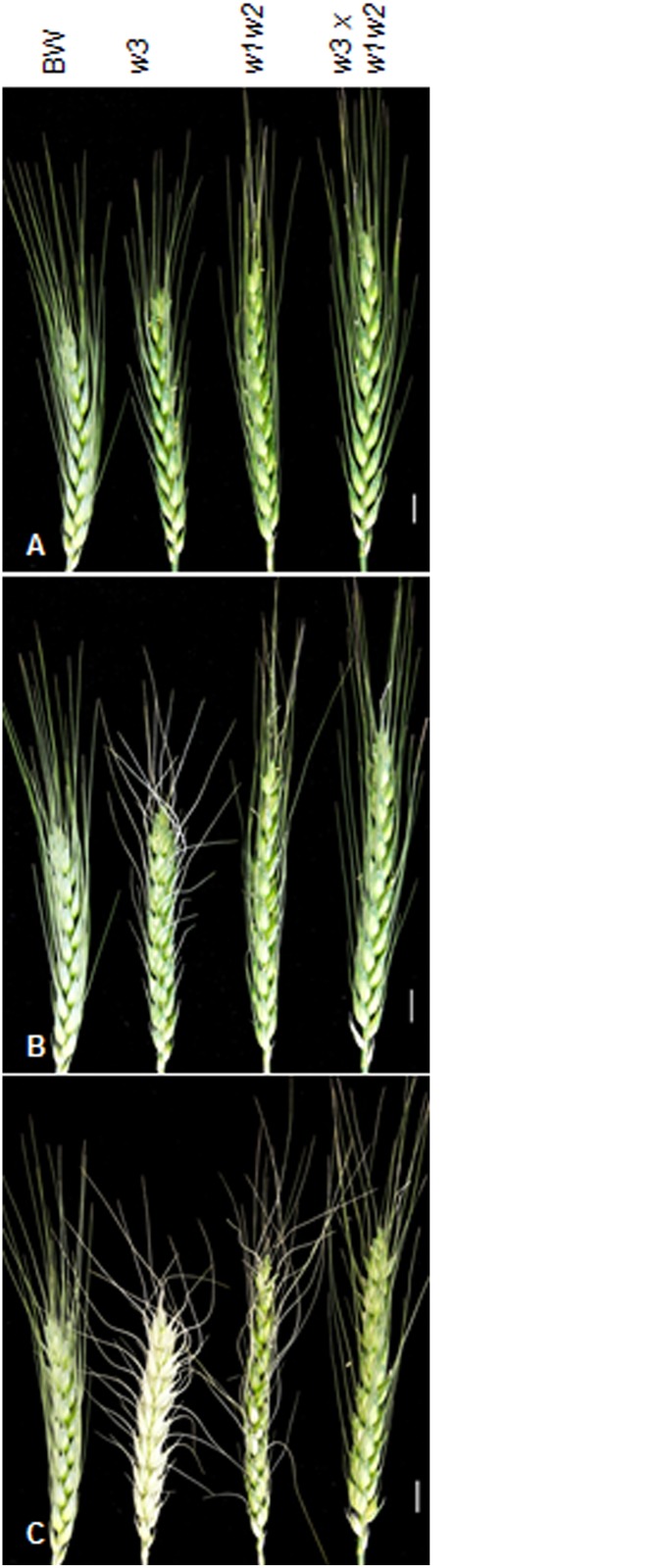
Spikes responses to dehydration. Spike of BW (*W1W1W2W2W3W3*), *w3* mutant (*W1W1W2W2w3w3*), *w1w2* double recessive line (*w1w1w2w2W3W3*), and the F_1_ hybrid between *w3* and *w1w2* (*W1w1W2w2W3w3*) at 0 (***A***), 6 (***B***) and 12 h of dehydration (***C***). The designations are indicated on the top. The scale bars indicate 1 cm.

Difference in water-loss rate was also found in spikes in relation to glaucousness. Six hours after detachment, top awns of the *w3* mutant started to dry (Fig 6B). Twelve hours after detachment, spike of *w3* mutant completely discolored and dried out; awns of *w1w2* double recessive line were twisted and some glumes discolored, but BW and F_1_ between *w3* mutant and *w1w2* stayed in normal shape (Fig 6C). This indicates that *W3* played an important role in preventing non-stomatal transpiration.

### Wax Composition

We extracted wax from the flag-leaf sheath at stage F10.5.1 and comparatively profiled the wax composition of BW and the *w3* mutant by GC-MS. Ninety-two wax molecules were identified, two thirds of which were trace and could not find a match in the National Institute of Standards and Technology database. These minors and unknowns account for ~10% total wax extract in BW, which had similar abundance in *w3* mutant.

**Fig 7:**
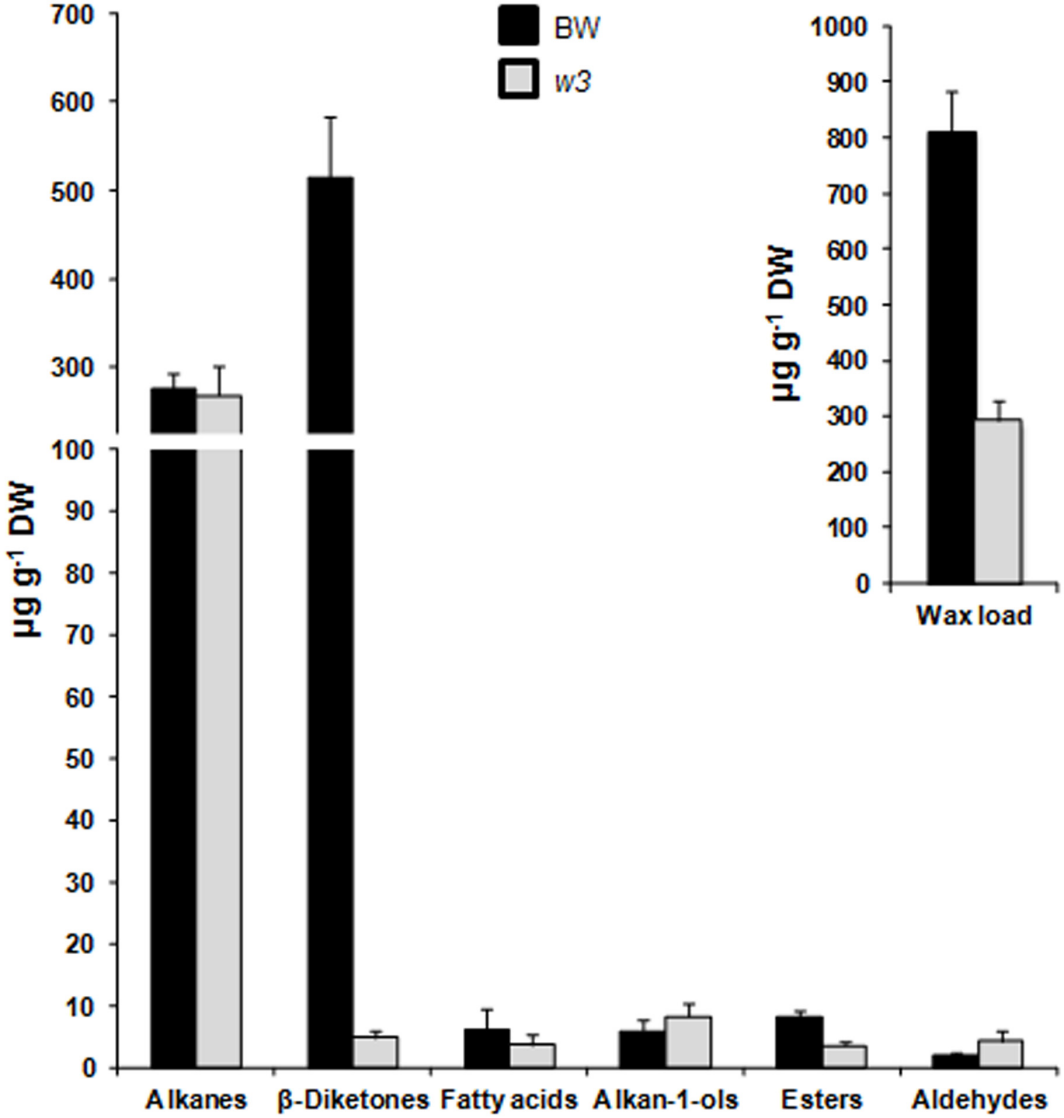
Wax composition of BW and *w3* mutant. Total wax load and content of alkanes, β-diketones, primary alcohols (alkan-1-ols), aldehydes, wax esters, and fatty acids of the flag leaf sheaths were measured by GC-MS. The numbers on the y-axes indicate average content expressed as μg per g dried tissue (dry weight, DW). The error bars indicate standard deviation of the mean estimated from five biological replicates.

**Fig 8:**
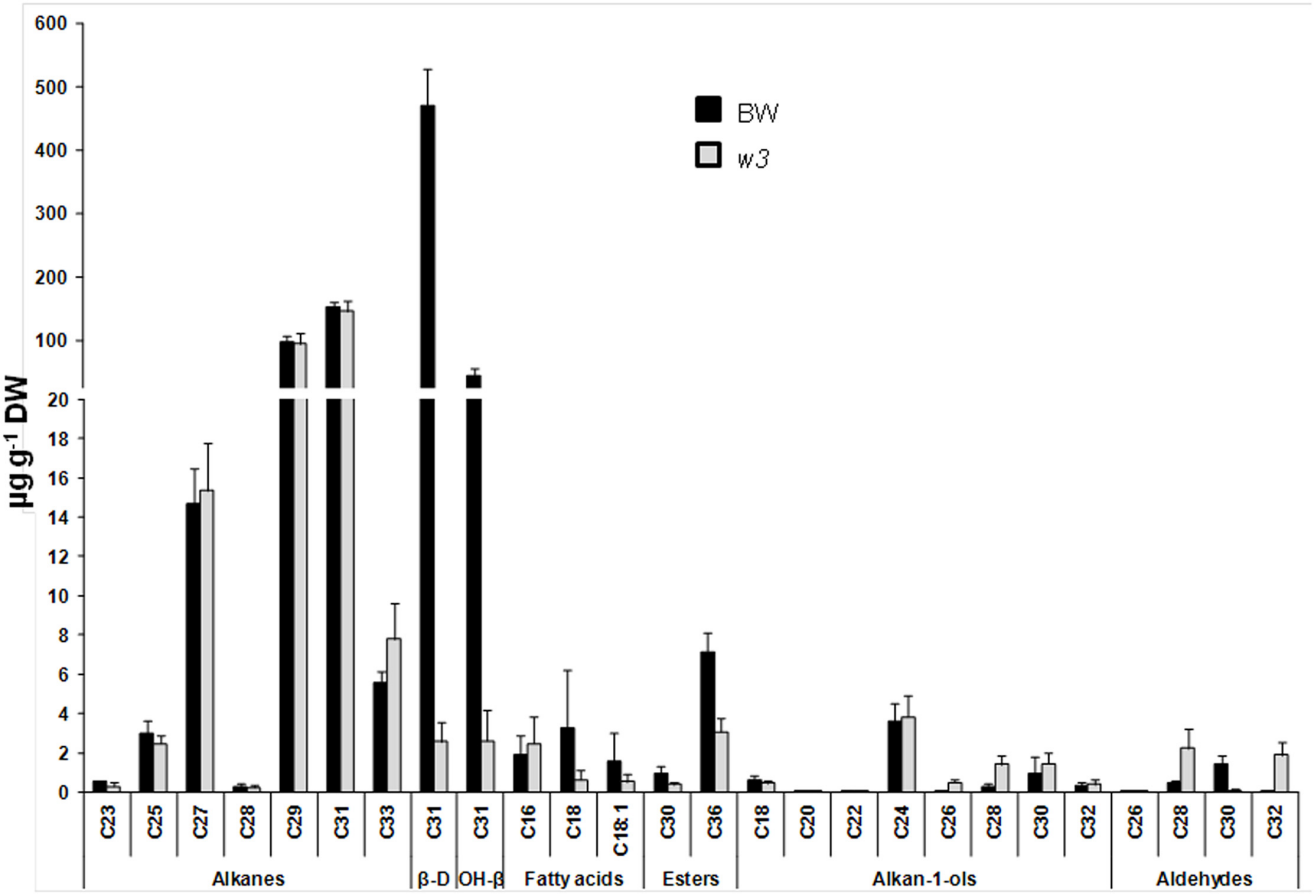
Variation of wax homologues between BW and *w3* mutant. Carbon atom numbers of alkanes, β-diketones, primary alcohols (alkan-1-ols), fatty acids, wax esters, and aldehydes are indicated on the x-axes. Their contents are indicated on y-axes as μg per g dried tissue (dry weight, DW). The error bars indicate standard deviation of the mean calculated from five biological replicates. β-D, β-diketone; and OH-β, hydroxy-β-diketones.

We analyzed the distribution patterns of major wax species in BW and *w3* mutant. In BW, two major wax components, diketones and alkanes account for 63.3% and 34.0% of the total wax load, respectively. Remaining 2.7% are wax esters (1.0%), fatty acids (0.8%), primary alcohols (0.7%) and aldehydes (0.2%). Dramatic differences were found in total wax load and wax composition between BW and *w3* mutant (Fig 7). Compared to BW, *w3* mutant lost 64% of the total wax (*P* = 5 x 10^−7^; Fig 7). While alkanes were maintained unchanged (*P* = 0.68113; Fig 7), β-diketones reduced to 1% in *w3* mutant (*P* = 1 x 10^−8^; Fig 7). As a result, alkanes account for over 90% of the total wax load of *w3* mutant (Fig 7). Two types of diketones, β-diketone and hydroxy-β-diketones, were detected. Hydroxy-β-diketones account for 8.5% of the total β-diketones in BW. As a result, BW had a hydroxy-β-diketones to β-diketone ratio (OH-D/β-D) of 0.0925. In *w3* mutant, β-diketone and hydroxy-β-diketones did not reduce proportionally: 181-fold reduction in β-diketone (*P* = 1 x 10^−7^), but 16-fold reduction in hydroxy-β-diketones (*P* = 0.00002; Fig 8). Two hydroxy-β-diketone isomers, 8- and 9-hydroxy hentriatane-14,16-dione, were detected. The 8-isomer reduced ~14-fold (*P* = 2 x 10^−5^) and the 9-isomer reduced ~22-fold (*P* = 0.00001) in *w3* mutant. Therefore, the hydroxy-β-diketones content was roughly equal to that of β-diketone in *w3* mutant with an OH-D/β-D of 1.014 (Fig 8), indicating a ~11-fold increase as compared to BW (*P* = 0.00695). This suggests a differential effect of the *w3* mutation on biosynthesis of β-diketone and its hydroxylation.

In addition to β-diketones, wax ester content, especially octadecanoic acid octadecyl ester (C_36_), was significantly decreased in *w3* mutant (*P* = 0.00002; Figs 7 and 8). Contrary to diketones and wax esters, total aldehyde was significantly increased in *w3* mutant (*P* = 0.01026; Fig 7). With regard to specific carbon length homologues of aldehydes, significant increase of C_28_ (*P* = 0.00309) and C_32_ (*P* = 0.00008) homologues and significant reduction of C_26_ (*P* = 0.00485) and C_30_ (*P* = 0.00008) homologues was observed in *w3* mutant (Fig 8). Although no significant difference was found between BW and *w3* mutant in the total primary alcohol and alkane content (Fig 7), changes were detected in some homologues of these wax species: C_20_ (*P* = 0.01215) alcohol was reduced in *w3* mutant, but C_22_, C_26_ and C_28_ alcohols (*P* < 0.00036) and C_33_ alkane (*P* = 0.02579) were significantly increased in *w3* mutant (Fig 8).

### Transcription of Cuticle Genes

**Fig 9:**
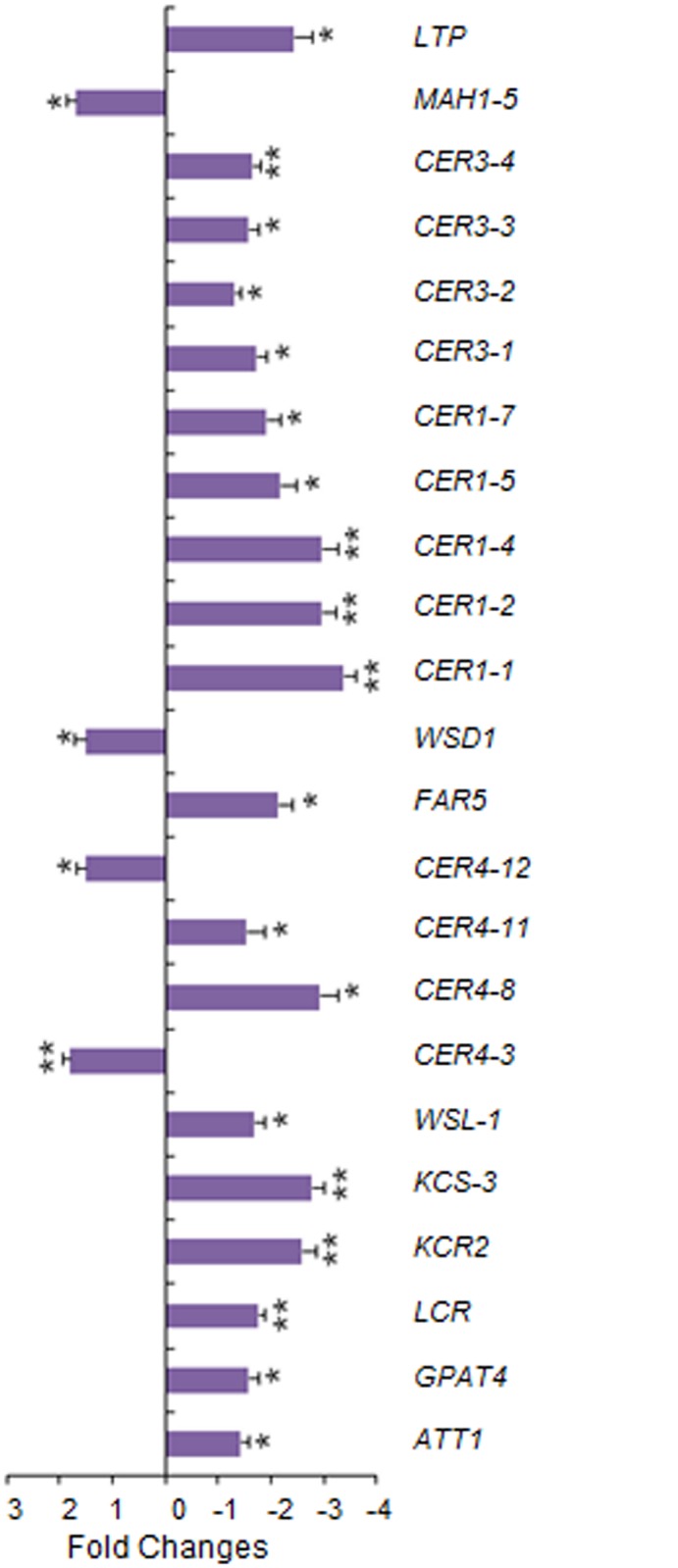
Transcriptional changes of wax genes in *w3* mutant against BW. Genes with significant fold changes are depicted. The error bars represent standard deviation of the mean fold-change of mRNA levels calculated from four biological replicates. Asterisks indicate that the difference is significant at *P*< 0.05 (*) or at *P* < 0.01 (**). Expression data for all genes analyzed are listed in S2 Table.

To gain insights into the *W3*-dependent genetic pathways, we profiled expression of 72 wheat wax candidate genes belonging to six categories [37] in the flag-leaf sheath of BW and *w3*-BW by qPCR. Of these 72 wax genes, five are responsible for cutin biosynthesis, 17 for fatty acyl elongation, 17 for VLCFA reduction and wax esterification, 21 for VLCFA decarbonylation, seven for wax transport and five for wax regulation (S2 Table). Result showed that expression of 19 genes was significantly down regulated and four up regulated (Fig 9). Of these 19 down-regulated genes, nine genes were down regulated more than twofold. These include five *CER1* members, *FAR5*, *KCR2*, *KCS-3* and *LTP* (Fig 9). Of the five down-regulated *CER1* genes, *CER1-1* showed greatest fold change, *i*.*e*. 3.4-fold down-regulation. Expression of four *CER3* genes reduced only ~1.5-fold though they may also be involved in decarbonylation via interaction with *CER1* gene products. By contrast, expression of *CER4-3*, *CER4-12*, *MAH1-5* and *WSD1* increased ~1.5-fold (Fig 9).

## Discussion

Glaucousness is one of the most eye-catching traits and has been used as a morphological marker in wheat genetic studies for ~80 years (reviewed in [30]). Its adaptive value in improving crop tolerance to drought and heat was recognized in 1980s [40, 41], and the climate change resumed interest in this trait more recently [36, 37, 42, 43]. Nevertheless, basic research of glaucousness in wheat lagged behind compared to other cereal crops, such as barley, a distant relative of wheat. One of the major reasons for this is lack of nonglaucous mutants in polyploid wheat. In this study, we identified a new β-diketone-deficit wax mutation *w3* and investigated the function of β-diketone as a major wax component in drought tolerance. Molecular characterization of *w3* mutant also shed light on diketone biosynthesis.

### Wax Loci

In barley, a diploid species, 79 wax loci have been identified by 1,580 induced *eceriferum* mutants [44], of which 53 mutant loci were localized to the all 7 chromosomes [45]. Chromosome 2H, which is homoeologous to wheat chromosomes 2A, 2B and 2D, carries 11 wax loci, of which *cer-c*, *cer-q*, *cer-u*, *cer-v* and *cer-zt* are located on the short arm and *cer-g*, *cer-n*, *cer-yb* and *gsh5* (*cer-s*) on the long arm. In contrast, only four wax loci, *W1*, *W2*, *Iw1* and *Iw2* have been identified in hexaploid wheat with *W1* and *Iw1* located on 2BS and *W2* and *Iw2* on 2DS [30]. In polyploid wheat, mutation rate are 5- to 10-fold higher than diploid species due to genetic redundancy [46, 47], but the mutant phenotypes are often masked by the wild type alleles of the homoeologous loci. The *W1* and *W2* genes are functionally redundant in forming glaucousness, but they differ in chromosomal locations [30] and expression profiles [37], suggesting a possibility that they are paralogous and their orthologs in the homoeologous chromosomes are silenced, deleted, neo- or sub-functionalized, which is an important feature of polyploid evolution [48]. In the present research, we showed *W3* is located on chromosome arm 2BS and complements with *W1* and *W2* for β-diketone production. Different from *W1* and *W2*, homoeologs of *W3* in the A and D genomes of polyploid wheat lost their function in glaucousness formation. This makes *W3* more tractable for genetic manipulation and molecular study of wheat wax.

In addition to BW, a single functional *W3* locus is present in CS-TDIC 2B (Table 1), probably also in CS. But it was previously not detected in the CS aneuploids, including nullisomics, nulli-tetrasomics (NT) and deletion lines. This failure was because *W3* is linked with *W1*. Deletion of either one causes the nonglaucous phenotype and prevents identification of another wax locus supposing that CS does not carry the *W2* allele. We examined the 42 NT lines and found that only N2B-T2A and N2B-T2D were nonglaucous. We also examined all eight homozygous deletion lines available for chromosome arm 2BS (2BS-1, -2, -3, -5, -6, -10,-12 and -14) and found that all of them were nonglaucous. These results confirmed that *W1* is located in the distal region of the chromosome arm [30], which prevents to identify additional wax loci on same chromosome arm although several wax loci located on homoeologous chromosome arm 2HS of barley [45]. No segregation in the F_2_ populations derived from the crosses between the *w3* mutant and 2BS deletion lines suggests that both *W1* and *W3* are located in the distal bin. The *w3* mutants may already exist in other wheat cultivars besides BW, but they are assumed to be the *w1* mutant. Therefore, allelism test is essential for identifying new wax gene loci in addition to marker linkage mapping. We are mapping the *W1* and *W3* loci, and the closely linked molecular markers will provide more detailed information on the organization of these wax loci on 2BS.

The *w3* mutation is independent of transgene insertions (S1 Fig), suggesting that it could have been induced by tissue culture process during transformation. It is well known that *in vitro* tissue culture generates genomic stress, reprograms cell development and causes genetic and epigenetic changes [49]. Some of these changes inherit as morphological or physiological mutants. Spontaneous mutation of other origin, however, cannot be excluded because we recently identified several other morphological mutants in BW, which did not go through tissue culture.

### *W3* Effect on Biosynthesis of β-Diketones

Like the wax genes *W1* and *W2* [37], *W3* is essential for biosynthesis of β-diketones, but had little effect on biosynthesis of alkanes. This supports the proposal that β-diketones are synthesized through a genetic pathway different from VLCFA synthesis and associated alkane-forming decarbonylation pathways although both β-diketones and alkanes are odd-carbon waxes [26]. Two biosynthetic pathways have been proposed for β-diketone biosynthesis: condensation of two C_16_ β-ketoesters via a “biological Claisen reaction” [50] and a modified elongation reaction on a selectively protected β-keto acid precursor [51, 52]. Both proposals lack molecular evidence although the latter was based on biochemical analysis of the substrate and inhibitor specificity [26], wax metabolite profiling and genetic analysis of the barley *cer-c*, *cer-q* and *cer-u* mutants [51]. In the both proposed models, a decarboxylative reaction was included. The *cer-q* and *cer-c* mutations impair the keto-protected chain elongation and lead to C_13_ and C_15_ alkan-2-ol formation [51, 52]. These secondary alcohols, products of β-keto fatty acid decarbonylation, were not detected either in *w1w2* [37] or the *w3* mutant in this research, suggesting that these wheat wax production genes act in upstream of the pathway or β-diketone is synthesized through the “biological Claisen reaction” in wheat [50]. qPCR assays showed that transcription of three FAE genes, five *CER1* and four *CER3* homologs was significantly down-regulated in *w3* mutant (Fig 9; S2 Table). In Arabidopsis, CER1 and CER3 are involved in VLCFA decarbonylation [11]. Because alkane content remained at the same level in *w3* mutant as in the wild type, these decarbonylation genes were not involved in alkane synthesis, but probably participated in the β-diketone-forming decarboxylative reaction. Considering that decarbonylation is the last step of β-diketone synthesis, transcription of these *CER1* and *CER3* homologs is possibly regulated either directly by *W3* or by a substrate-mediated feed-forward loop.

The hydroxy β-diketone isomers are derived from β-diketone by hydroxylation [27], hence their changes in *w3* mutant are expected to be proportional. Contrary to this expectation, *w3* mutant had a much higher HO-D/β-D ratio compared to BW. This result suggests that hydroxylation reaction enzymes in the BW background are less sensitive to depletion of the substrate, i.e. β-diketone, or *W3* promotes synthesis of β-diketone but suppresses its hydroxylation. In agreement with the latter hypothesis, expression of *MAH1-7*, a homolog of *MAH1* that is responsible for midchain alkane hydroxylation in Arabidopsis [10], increased 1.5-fold in the *w3* mutant (Fig 9; S2 Table). In another research, we found that interaction between wax production genes *W1* and *W2* is required for biosynthesis of hydroxy-β-diketones and that up-regulation of *MAH1-8* paralleled with β-diketone hydroxylation in the *W1W2* double dominant genotype [37]. It would be interesting to see if *W3* interacts with *W1* and *W2* by combining their loss-of-function mutations in the same genetic background and measuring their effect on the hydroxy β-diketone isomers.

In addition to FAE, decarbonylation and hydroxylase genes, expression of three cutin biosynthetic genes, *FAR5* and the putative wax transporter *LTP* was also down regulated in the *w3* mutant (Fig 9; S2 Table). It is hard to imagine how a single-gene mutation affects expression of genes functioning in five cuticle pathways. In the model plant Arabidopsis, mutation in wax regulator *SHN* of the AP2/EREBP family altered property of both wax and cutin and expression of genes in multiple pathways [53–55]. We measured expression of five known wax regulator genes, but none of them changed significantly (S2 Table). Accordingly, one possibility would be that *W3* encodes a wax regulator that orchestrates transcription of the biosynthetic and transporter genes and those acting at the upstream of β-diketone pathway. This may explain the mutant effect on other wax species, such as wax esters, C_33_ alkane, C_22_, C_26_ and C_28_ alcohol, and aldehydes (Fig 8). An alternative and less likely scenario would be that *W3* targets on the initial step of diketone biosynthesis and the expression of these 20 genes are regulated by substrate-mediated feed-forward loops or secondary responses as discussed earlier. In either case, our results suggest a possibility that β-diketone pathway in Triticeae uses some *CER1* and *CER3* homologs for decarbonylation and *MAH1* homologs for hydroxylation.

### β-Diketones and Glaucousness

Cuticular waxes from the upper parts of adult Triticeae plants can be divided into two types according to their major constituents: primary alcohol-rich and β-diketone-rich wax. The diploid wheat *T*. *monococcum* and *T*. *urartu* belong to the former and polyploid wheat *T*. *turgidum* and *T*. *timopheevii* to the latter type [56, 57]. Analyses of NG mutants of barley [27] and wheat [28, 36, 37, 58, 59] indicated that β-diketones play an important role in glaucousness development. Wax profiling of the wheat wax gene NILs identified three types of waxes from the flag leaf sheaths: β-diketone-rich wax from the glaucous NILs carrying *W1w2*, *w1W2* or *W1W2*, alkane-rich wax from the nonglaucous NIL *w1w2*, and alcohol-rich wax from the nonglaucous NILs carrying the wax inhibitor *Iw1* or *Iw2* [37]. In BW, β-diketone and hydroxy-β-diketones account for 63% of the total wax load. In the *w3* mutant, β-diketones reduced ~100-fold. At the same time, wax crystal tubes were almost invisible in the flag-leaf sheath (Fig 4B). This confirms the role of β-diketones in glaucousness determination.

Next to β-diketones, alkanes, mainly C_29_ and C_31_ homologues, constitute the second major wax component, accounting for 34% of the total wax in BW. Although only significant net change was found in C_33_ alkane (Fig 8), proportion of the whole alkanes was increased to 90% due to depletion of the β-diketones. Significant differences were also observed between BW and *w3* mutant in aldehydes, primary alcohols, and wax esters, but they had very low abundance, less than 3% of total wax in BW, suggesting that they are less likely to contribute to the mutant phenotype.

### β-Diketones and Abiotic Stress

Early physiological studies in cereal crops showed that glaucousness increased grain yield by >7% in barley [60] and wheat [40, 41]. More recent experiments performed in Mexico, where no rainfall was received during the growing cycle, showed glaucousness significantly contributed to grain yield even in irrigated condition [42].

Our recent research using the *W1*, *W2*, *Iw1* and *Iw2* NILs indicated that glaucousness significantly reduced cuticle permeability expressed as water loss and chlorophyll efflux. Difference in cuticle permeability was also found among the glaucous NILs, which differed in the hydroxy-β-diketones, and suggested a role of these hydroxy isoforms in drought tolerance [37]. In the present research, we showed that the *w3* mutation impairs the biosynthesis of β-diketone and further deplete the hydroxy forms. At the same time, the *w3* mutation significantly increased water loss and chlorophyll leaching (Fig 5). In the spike, *w3* mutant showed even faster water loss than the *w1w2* double recessive plant (Fig 6). One explanation is that the *w3* mutant lost 99% of β-diketones (Fig 7), but they reduced 92% in the *w1w2* NIL [37]. In addition to cuticular waxes, cutin proper plays an even greater role in protecting the non-controllable water loss as recently demonstrated in Arabidopsis [61] and barley [62]. In the *w3* mutant, three of the five cutin genes assayed showed ~1.5-fold down-regulation (Fig 9; S2 Table). Transmission electron microscopic inspection of cutin organization in BW and *w3* mutant may provide more insights into the effect of the *w3* mutation on cutin foundation organization.

Another important function of glaucousness is to reflect extra irradiation [1]. In this respect, β-diketones greatly contribute to heat tolerance. The glaucousness can reduced the photosynthetic temperature by up to 0.7°C in the drought-stressed field [41]. We observed that well-watered *w3* mutant plants died prematurely in the later spring seasons of 2010 and 2011 in a temperature poorly-controlled greenhouse room (Fig 1C). This did not occur in the fall and winter seasons, suggesting that loss of β-diketones in *w3* mutant increased its susceptibility to the high temperature. Preliminary result from field experiment in 2014 showed that *w3* mutation significantly reduced 1000-kernel weight (Wanlong Li and Karl Glover, unpublished data). Detailed physiological experiments need to be conducted using growth chambers with well-controlled temperatures and relative humidity to shed light on how the *w3* mutation affects the thermal characteristics of adult wheat plants.

## Materials and Methods

### Plant Materials and Growing Conditions

Transgenic plant #056 was generated in BW by bombardment transformation of RNAi construct pVGS193 (Bi C, Trick HN, Gill BS and Li W, unpublished), which was developed using pANDA, a Gateway-based RNAi vector [38]. Other plant materials are listed in S3 Table with their accession numbers, wax genotypes, wax phenotypes and sources of seeds. The crosses were made by manual emasculation and pollination. All the plants were grown in 4”x4” pots containing Sunshine^®^ potting mix #3 (Sun Gro Horticulture, Agawam, MA, USA) supplemented with Multicote^®^8 Controlled-Release Fertilizer (Haifa, Altamonte, FL, USA) in a greenhouse. Temperature in the greenhouse was set as 22° at day and 17° at night, and day length was 16 h.

### Markers Genotyping and Linkage Mapping

Genomic DNA was isolated following the procedure described by [63]. Polymerase chain reaction (PCR) was conducted in 10 μl containing ~40 ng genomic DNA, 250 μM dNTPs, 200 nM primers, 0.5 unit of Taq polymerase, and 1× Green Go-Taq Reaction Buffer (Promega, Madison, WI, USA) and implemented on 9700 dual thermocyclers (ABI), which were programmed as 94°C for 5 min, then 40 cycles of 94°C 20 s, 55–60°C 30 s and 72°C 1 min 20 s, and finally 7 min at 72°C. The PCR products were separated by 1.5% agarose or 6% polyacrylamide gel electrophoresis.

We used MAPMAKER3.0 [64] for determining the order of marker and gene loci and the Kosambi mapping function for converting the recombination into genetic distance in terms of centi-Morgan (cM) [65]. An LOD score of 3.0 is applied to all marker loci.

### Microscopy

For stoma counting and aperture observations, both sides of flag leaf sheaths were coated with 10% cellulose acetate dissolved in acetone using a paint brush. Air-dried for 5 min, the cellulose film was carefully peeled and the imprinted slides were observed under a light microscope at a magnification of 10x20. The fresh cuticle samples of flag leaf sheaths were sputtered with gold powder using the CrC-150 Sputtering System and inspected using a Hitachi S-3400N SEM (Hitachi, Tokyo, Japan).

### Cuticle Trait Measurements

At Feekes’ stage 10.5.1 (F10.5.1) when wheat plants flower, flag leaf sheathes were detached and immersed in 30 ml 80% ethanol in a 50-ml falcon tube. The capped tube was agitated at 50 rpm on an orbital shaker. A 150-μl aliquot was removed at a two-hour time interval for measuring the absorbance at 648 nm and 664 nm on Synergy 2 Multi-Mode Microplate Reader (Biotek, Winooski, VT, USA). The sample was returned to the same tube after measurement. Six replicates of extraction were conducted each from an individual of the same line. Two measurements were performed for each extraction and their values were averaged for subsequent calculation. The sheaths were also subjected to air-drying at room temperature with 45% relative humidity. Their weight was measured at a one-hour time interval for 12 consecutive hours to evaluate the water loss rate using an AB54-S/FACT analytical balance (Mettler Toledo, Columbus, OH, USA) with an accuracy of ±0. 0001 g.

### Wax Extraction and Metabolite Analysis

One flag leaf sheath was detached at stage F10.5.1 and immersed in 10 ml HPLC-grade chloroform containing 2 μg tetracosane as an internal reference, which is not present in plant wax, and gently agitated for 1 min, the tissue was rinsed in another tube containing 5 ml chloroform. Two extracts were merged and purified by filtration with Iso-Disc PTFE-13-2 filter (Sigma, St Louis, MO, USA). For each line, five biological replicates were extracted separately. Purified wax extract was dried under a nitrogen stream. Wax silylation, gas chromatography–mass spectrometry (GC-MS) profiling, and substance identification were performed at the W.M. Keck Metabolomics Research Laboratory of Iowa State University (Ames, IA, USA) on a fee-for-service basis. In brief, the dried wax extract was dissolved in 100 μl of BSTFA+TCMS (N,O-Bis(trimethylsilyl)trifluoroacetamide with 1% trimethylchlorosilane) and derivatized at 80°C for 30 minutes. The solution is dried under a stream of nitrogen and the residue is reconstituted in 50 μl chloroform and subjected to GC-MS analysis. GC-MS analysis was performed with an Agilent 6890 GC interfaced to a 5973 mass spectrometer. The HP-5ms column (30 m x 0.25 mm i.d. coated with a 0.25 μm film, Agilent Technologies) was used, and temperature gradient was programmed from 150 to 320°C at 5°C/min with helium flow rate at 1.0 ml/min. Operating parameters for MS were set to 70 eV of ionization voltage and 280°C of interface temperature.

### Quantitative RT-PCR

Of the 72 quantitative RT-PCR (qPCR) primer pairs used, 64 were adopted from Zhang et al [37] and eight pairs from Kosma et al. [66] together with their gene name designations. At stage F9.0 when the flag leaf is fully emerged from the whorl and flag leaf sheath is rapidly elongating, the flag leaf sheath was collected and immediately frozen in liquid nitrogen and used for RNA isolation using Trizol reagent (Thermo Fisher Scientific Inc., Waltham, MA, USA) following the manufacturer’s instruction. Four biological replicates were used. After evaluating RNA integrity in agarose gel and quantification with Nanodrop ND-1000 (Thermo Scientific, Waltham, MA, USA), 1 μg total RNA was used for reverse transcription using QuantiTect Reverse Transcription Kit (Qiagen, Valencia, CA, USA). Approximately 5 ng cDNA was used as template for qPCR, which was performed on ABI 7900HT High-Throughput Real-Time Thermocycler (Thermo Fisher Scientific Inc.) using the iTaq™ SYBR^®^ Green Supermix with ROX (Bio-Rad, Hercules, CA, USA). Two technical replicates were included for each biological replicate. Melt curve analysis was conducted to determine the amplified specificity of PCR products. *TaRPII36* was used as the internal reference [37] and relative transcript abundance in the RNA samples was quantified using the 2^-ΔΔCt^ method as described by [67].

### Data Analysis and Statistics

Wax load, components, chlorophyll efflux, water loss, and wax gene transcription were measured from four or more biological replicates. The means, standard deviations and *P* values were estimated using Microsoft^®^ Excel functions. Student’s t-tests were performed to evaluate statistical significance of the differences between BW and the NG mutant. Pearson's chi-squared test was calculated for the deviation of F_2_ segregation from the expected ratios. The cut-off for statistical significance was set to *P*-value ≤ 0.05.

## Supporting Information

S1 Fig PCR screening of transgenes in the nonglaucous F_2_ segregants. The upper band (arrow) was detected using a primer pair targeting on *gus-nos* junction (5’- CATGAAGATGCGGACTTACG-3’ and 5’- GCGCGCTATATTTTGTTTTC-3’). The lower band (arrow) was detected using a primer pair targeting on *Ubq-bar* junction (5’- GAAGTCCAGCTGCCAGAAAC-3’ and 5’- GCACCATCGTCAACCACTAC-3’). The designation of plant lines are indicated above the picture. BW, Bobwhite; NG1, nonglaucous mutant line 1; NG2, nonglaucous mutant line 2; CS, Chinese Spring. NG1, the female parent of the F_2_ population, carries the bar and RNAi transgene; NG2 is negative for either of them. BW and CS are the negative controls.
(TIF)

S1 Table Genotyping of Bobwhite and w3 mutant line NG2 with SSR markers located in distal end of wheat chromosome arms (DOCX)

S2 Table Expression of wax genes in the w3 mutant against BW. (DOCX)

S3 Table A list of plant materials used. (DOCX)

## Abbreviations

BW: Bobwhite
CS: Chinese Spring
CS-TDIC 2B: Chinese Spring-*T*. *turgidum* subsp. *dicoccoides* 2B substitution line
β-D: β-diketone
CER: ECERIFERUM
FAE: fatty acyl elongase
FAR: fatty acyl-CoA reductase
GL: GLOSSY
KCR: β-keto acyl-CoA reductase
KCS: β-keto acyl-CoA synthases
LTP: lipid transfer protein
MAH: midchain alkane hydroxylase
NIL: near isogenic line
NG: nonglaucous
NT: nulli-tetrasomic
OH-β: hydroxy β-diketone
qPCR: quantitative real time PCR
SEM: scanning electron microscope
VLCFA: very long chain fatty acid
WSD: Wax Ester Synthase/Acyl-Coenzyme A:Diacylglycerol Acyltransferase
