# Supplementary figures and images for: W3 Is a New Wax Locus That Is Essential for Biosynthesis of β-Diketone, Development of Glaucousness, and Reduction of Cuticle Permeability in Common Wheat
Source: PLoS One. 2015 Oct 15;10(10):e0140524. doi: 10.1371/journal.pone.0140524 (PMC4607432; doi:10.1371/journal.pone.0140524)

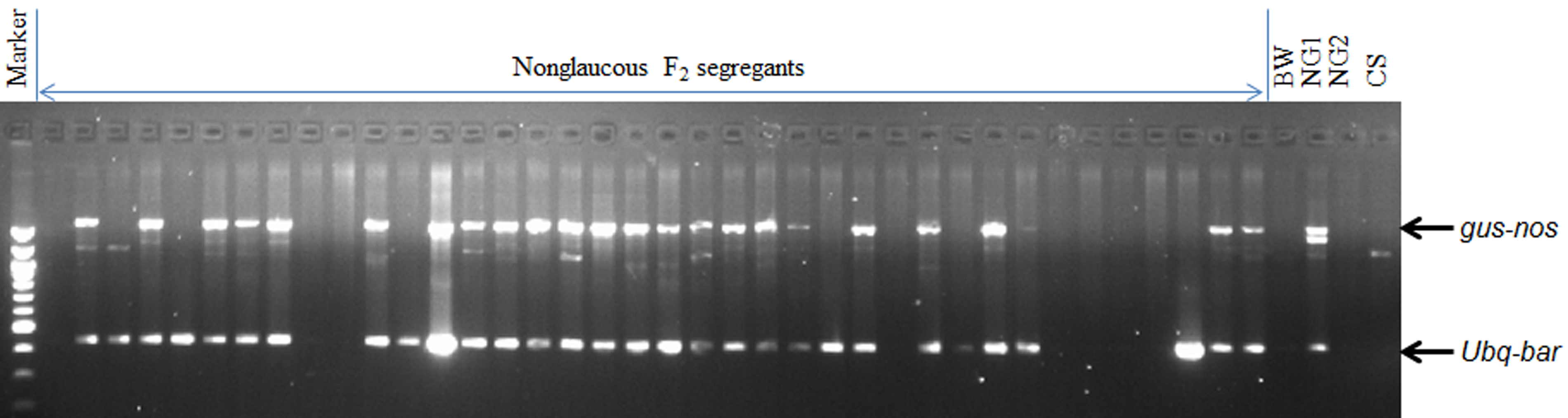

Supplement: S1 Fig — The upper band (arrow) was detected using a primer pair targeting on gus-nos junction (5’- CATGAAGATGCGGACTTACG-3’ and 5’- GCGCGCTATATTTTGTTTTC-3’). The lower band (arrow) was detected using a primer pair targeting on Ubq-bar junction (5’- GAAGTCCAGCTGCCAGAAAC-3’ and 5’- GCACCATCGTCAACCACTAC-3’). The designation of plant lines are indicated above the picture. BW, Bobwhite; NG1, nonglaucous mutant line 1; NG2, nonglaucous mutant line 2; CS, Chinese Spring. NG1, the female parent of the F2 population, carries the bar and RNAi transgene; NG2 is negative for either of them. BW and CS are the negative controls. (TIF) [file pone.0140524.s001.tif]
