# Supplementary material for: W3 Is a New Wax Locus That Is Essential for Biosynthesis of β-Diketone, Development of Glaucousness, and Reduction of Cuticle Permeability in Common Wheat
Source: PLoS One. 2015 Oct 15;10(10):e0140524. doi: 10.1371/journal.pone.0140524 (PMC4607432; doi:10.1371/journal.pone.0140524)
Supplement: S1 Table — (DOCX) [file pone.0140524.s002.docx]

**S1 Table.** Genotyping of Bobwhite and w3 mutant line NG2 with SSR markers located in distal end of wheat chromosome arms

| Chromosome arm | Markers | BW | *w3* |
| --- | --- | --- | --- |
| 1AS | gdm33 | + | + |
| 1AL | gwm99 | + | + |
| 1BS | gwm608 | + | + |
| 1BL | gwm140 | + | + |
| 1DS | gwm147 | + | + |
| 1DL | barc62 | + | + |
| 2AS | cfd36 | + | + |
| 2AL | gpw4456 | + | + |
| 2BS | wmc764 | + | + |
| 2BL | wmc602 | + | + |
| 2DS | barc124 | + | + |
| 2DL | gwm301 | + | + |
| 3AS | wmc11 | + | + |
| 3AL | gwm480 | + | + |
| 3BS | barc75 | + | + |
| 3BL | gwm340 | + | + |
| 3DS | cfd35 | + | + |
| 3DL | barc71 | + | + |
| 4AS | gwm4 | + | + |
| 4AL | wmc219 | + | + |
| 4BS | wmc125 | + | + |
| 4BL | wmc617 | + | + |
| 4DS | wmc285 | + | + |
| 4DL | gwm609 | + | + |
| 5AS | barc10 | + | + |
| 5AL | gwm410 | + | + |
| 5BS | cfd5 | + | + |
| 5BL | wmc258 | + | + |
| 5DS | wmc233 | + | + |
| 5DL | gwm654 | + | + |
| 6AS | gwm459 | + | + |
| 6AL | wmc59 | + | + |
| 6BS | gwm613 | + | + |
| 6BL | barc134 | + | + |
| 6DS | cfd49 | + | + |
| 6DL | wmc773 | + | + |
| 7AS | gwm666 | + | + |
| 7AL | wmc809 | + | + |
| 7BS | wmc606 | + | + |
| 7BL | gwm344 | + | + |
| 7DS | gwm350 | + | + |
| 7DL | cfd175 | + | + |
