# Supplementary material for: W3 Is a New Wax Locus That Is Essential for Biosynthesis of β-Diketone, Development of Glaucousness, and Reduction of Cuticle Permeability in Common Wheat
Source: PLoS One. 2015 Oct 15;10(10):e0140524. doi: 10.1371/journal.pone.0140524 (PMC4607432; doi:10.1371/journal.pone.0140524)
Supplement: S2 Table — (DOCX) [file pone.0140524.s003.docx]

**S2 Table.** Expression of wax genes in the w3 mutant against BW

| Genes | Fold change^1^ | Standard deviation | Significance ^2^ |
| --- | --- | --- | --- |
|  |  |  |  |
| Cutin biosynthesis |  |  |  |
| *ATT1* | -1.43 | 0.117943907 | * |
| *BDG* | 1.02 | 0.17189469 |  |
| *GPAT4* | -1.57 | 0.179778706 | * |
| *HTH1* | -1.03 | 0.164422743 |  |
| *LCR/CYP86A8* | -1.74 | 0.120366833 | ** |
|  |  |  |  |
| Fatty acyl elongation |  |  |  |
| *ACC1* | -1.20 | 0.174196518 |  |
| *CER10/ECR* | 1.06 | 0.165690523 |  |
| *FATB* | -1.33 | 0.325479031 |  |
| *GL8* | 1.03 | 0.150472825 |  |
| *KCR1* | -1.07 | 0.11624677 |  |
| *KCR2* | -2.59 | 0.251778961 | ** |
| *KCS1* | 1.25 | 0.131645737 |  |
| *KCS-1* | 1.04 | 0.150096274 |  |
| *KCS2/Daisy* | 1.28 | 0.20633764 |  |
| *KCS-2* | -1.16 | 0.169628173 |  |
| *KCS-3* | -2.75 | 0.241574258 | ** |
| *KCS-4* | -1.28 | 0.227384117 |  |
| *KCS-5* | -1.03 | 0.107044164 |  |
| *KCS6/CER6* | 1.06 | 0.227527501 |  |
| *LACS1* | -1.31 | 0.110935018 |  |
| *LACS3* | -1.09 | 0.149119638 |  |
| *WSL1* | -1.67 | 0.210725324 | * |
|  |  |  |  |
| Fatty acyl reduction |  |  |  |
| *CER4-1* | -2.64 | 0.336549267 |  |
| *CER4-2* | -3.41 | 0.361798082 |  |
| *CER4-3* | 1.82 | 0.15339212 | ** |
| *CER4-4* | 1.18 | 0.366507608 |  |
| *CER4-5* | -1.23 | 0.365702907 |  |
| *CER4-6* | 1.56 | 0.338991286 |  |
| *CER4-7* | -1.11 | 0.090655282 |  |
| *CER4-8* | -2.91 | 0.347516948 | * |
| *CER4-9* | -2.45 | 0.30427618 |  |
| *CER4-10* | -1.96 | 0.366604292 |  |
| *CER4-11* | -1.53 | 0.344907679 | * |
| *CER4-12* | 1.52 | 0.165080042 | * |
| *CER4-13* | -1.17 | 0.168615601 |  |
| *CER4-14* | -1.21 | 0.332292527 |  |
| *FAR2* | 1.65 | 0.367260557 |  |
| *FAR5* | -2.14 | 0.268154722 | * |
| *WSD1* | 1.51 | 0.230404897 | * |
|  |  |  |  |
| Decarbonylation |  |  |  |
| *CER1-1* | -3.35 | 0.250678079 | ** |
| *CER1-2* | -2.96 | 0.247055016 | ** |
| *CER1-3* | -1.07 | 0.366311558 |  |
| *CER1-4* | -2.95 | 0.298056283 | ** |
| *CER1-5* | -2.17 | 0.291387359 | * |
| *CER1-6* | 1.16 | 0.337060436 |  |
| *CER1-7* | -1.89 | 0.26198592 | * |
| *CER1-8* | 1.16 | 0.339207145 |  |
| *CER3-1* | -1.70 | 0.218366494 | * |
| *CER3-2* | -1.30 | 0.133324806 | * |
| *CER3-3* | -1.56 | 0.193426944 | * |
| *CER3-4* | -1.63 | 0.170061594 | ** |
| *CER3-5* | -1.33 | 0.261402985 |  |
| *MAH1-1* | -1.00 | 0.26852971 |  |
| *MAH1-2* | - | - |  |
| *MAH1-3* | 1.23 | 0.327198921 |  |
| *MAH1-4* | -1.04 | 0.16282284 |  |
| *MAH1-5* | 1.71 | 0.177251886 | * |
| *MAH1-6* | 1.52 | 0.352583165 |  |
| *MAH1-7* | 2.30 | 0.354219376 |  |
| *MAH1-8* | 1.28 | 0.190648593 |  |
|  |  |  |  |
| Transporters |  |  |  |
| *ABCG15* | 1.15 | 0.165506674 |  |
| *HvABCG31* | -1.26 | 0.178313699 |  |
| *LTP* | -2.44 | 0.32101536 | * |
| *LTP1* | 1.16 | 0.273274454 |  |
| *LTP4* | -1.23 | 0.161804017 |  |
| *WBC11/ABCG11* | 1.22 | 0.185852595 |  |
| *WBC15/ABCG15* | 1.27 | 0.139295286 |  |
| *WBC19/ABCG19* | -1.01 | 0.151599573 |  |
|  |  |  |  |
| Regulators |  |  |  |
| *CER7* | -1.03 | 0.158524167 |  |
| *MYB30* | -1.17 | 0.120963146 |  |
| *MYB96* | 1.34 | 0.26914691 |  |
| *OCL1* | 1.02 | 0.203872761 |  |
| *WIN1/SHN1* | -1.14 | 0.29456319 |  |

^1^ the minus "-" indicates down-regulation

^2^Asterisks indicate that the difference is significant at P< 0.05 (*) or at P< 0.01 (**)
