# Supplementary material for: W3 Is a New Wax Locus That Is Essential for Biosynthesis of β-Diketone, Development of Glaucousness, and Reduction of Cuticle Permeability in Common Wheat
Source: PLoS One. 2015 Oct 15;10(10):e0140524. doi: 10.1371/journal.pone.0140524 (PMC4607432; doi:10.1371/journal.pone.0140524)
Supplement: S3 Table — (DOCX) [file pone.0140524.s004.docx]

**S3 Table. A list of plant materials used.**

| Accessions | Description | Wax phenotype | Wax Genotype | Fraction length* | Sources |
| --- | --- | --- | --- | --- | --- |
|  | S165-*w1w2* | nonglaucous | *w1w1w2w2W3W3* |  | Kio Tsunewaki |
| TA9087 | Bobwhite (BW) | glaucous | *W1W1W2W2W3W3* |  | Bikram S. Gill |
|  | BW-NG1 | nonglaucous | *W1W1W2W2w3w3* |  | This research |
|  | BW-NG2 | nonglaucous | *W1W1W2W2w3w3* |  | This research |
| TA3008 | Chinese Spring (CS) | glaucous | *W1W1W2W2W3W3* |  | Bikram S. Gill |
| TA3460 | CS-DIC 2B | glaucous | *W1W1W2W2W3W3* |  | Bikram S. Gill |
| TA3266 | N2B-T2D** | nonglaucous | *─ ─ W2W2 ─ ─* |  | Bikram S. Gill |
| TA4518 L1 | 2BS-1 deletion line | nonglaucous | *─ ─ W2W2 ─ ─* | 0.53 | Bikram S. Gill |
| TA4518 L2 | 2BS-2deletion line | nonglaucous | *─ ─ W2W2 ─ ─* | 0.15 | Bikram S. Gill |
| TA4518 L3 | 2BS-3deletion line | nonglaucous | *─ ─ W2W2 ─ ─* | 0.84 | Bikram S. Gill |
| TA4518 L5 | 2BS-5 deletion line | nonglaucous | *─ ─ W2W2 ─ ─* | 0.79 | Bikram S. Gill |
| TA4518 L6 | 2BS-6 deletion line | nonglaucous | *─ ─ W2W2 ─ ─* | 0.56 | Bikram S. Gill |
| TA4518 L10 | 2BS-10 deletion line | nonglaucous | *─ ─ W2W2 ─ ─* | 0.16 | Bikram S. Gill |
| TA4518 L12 | 2BS-12 deletion line | nonglaucous | *─ ─ W2W2 ─ ─* | 0.81 | Bikram S. Gill |
| TA4518 L14 | 2BS-14 deletion line | nonglaucous | *─ ─ W2W2 ─ ─* | 0.83 | Bikram S. Gill |

* The position of deletion breakpoint was expressed as a fraction length value of the arm retained in the deletion chromosome.

**N2B-T2D is abbreviation of nullisomic 2B-tetrasomic 2D.
